# Effects of the COVID-19 Pandemic on the Budgetary Mechanism Established to Cover Public Health Expenditure. A Case Study of Romania

**DOI:** 10.3390/ijerph18031134

**Published:** 2021-01-28

**Authors:** Mihaela Onofrei, Elena Cigu, Anca-Florentina Gavriluta (Vatamanu), Ionel Bostan, Florin Oprea

**Affiliations:** 1 Faculty of Economics and Business Administration, Alexandru Ioan Cuza University, 22 Carol I, 700505 Iasi, Romania; onofrei@uaic.ro (M.O.); elena.chelaru@uaic.ro (E.C.); anca.vatamanu@mail.uaic.ro (A.-F.G.); foprea@uaic.ro (F.O.); 2 Faculty of Law and Administrative Sciences, Ștefan cel Mare University, Universitatii 13, 720229 Suceava, Romania

**Keywords:** COVID-19 pandemic, health expenditure, budgetary space, economic resilience, social distancing measures

## Abstract

The COVID-19 pandemic stressed the importance of understanding the sources of vulnerabilities that can lead to a financial crisis and highlighted the predominant impact on health systems. Firstly, the paper aims to conduct a retrospective analysis of the Romanian health care system, over the period of time 1985–2019, based on our own computed sustainability index for public health. Secondly, using the Gregory-Hansen cointegration method, we provide new evidence on the causal relationship between health expenditure and GDP for Romania over the period of time 1985–2017. Based on the retrospective analysis of the long-run co-movement between health spending and GDP, the study allows one to prospectively examine not only the effects of the COVID-19 pandemic on health care spending, but also to reveal the government’s fiscal position and vulnerabilities. Our results highlight the intergenerational costs related to the policy incoherence roadmap and regulatory fragmentation, stressing the importance of economic system resilience through fiscal diligence and the consolidation of the institutional context.

## 1. Introduction

The sharp spread of the COVID-19 pandemic in Europe, implicitly in Romania, and the increasingly stringent measures taken by the authorities to prevent harmful effects on citizens and to the economy, stressed the importance of understanding the sources of vulnerabilities that can directly affect health systems and the entire economic background of each country. Under the COVID-19 pandemic, countries strategies were targeted to ensure the sustainability of health care systems in dealing with limited intensive care unit (ICU) capacity and equipment [1], and to support the business economy by providing cash, loans and guarantees to compensate, as much as possible, for the losses incurred because of the lockdown effects (during and after). In this regard, the Stability and Growth Pact rules were suspended by recognition of a severe economic downturn in the European Union, determining also a relaxation of state aid rules in order to support the business sector during the lockdowns and later. The European Commission has approved loans to EU Member States up to 2% of GDP to pay the direct and indirect costs of health care and the prevention of COVID-19 without conditionality, with a maximum of 10 years average maturity. Other facilities for the EU countries were approved for restarting the economies of the EU and for protecting jobs or companies, under the form of loans to national governments or other non-reimbursable funds.

Meanwhile, literature and International Organizations are trying to provide information on COVID-19 policies nationwide and statistics, precisely in order to optimally identify sustainable public policies to combat the pandemic [2,3,4,5].

One of the major aims of this paper is to clearly highlight the status of the health system in Romania, explained with the help of a sustainability index for public health that is own computed. Another major aim of the paper is to provide new evidence on the causal relationship between health expenditure and economic growth for Romania using the Gregory-Hansen cointegration method. Secondly, the paper can be considered as an important contribution to the research field for Romania because, based on own computed sustainability index for public health, we evaluate the causal relationship between health expenditure and GDP.

This paper contributes to the existing literature in many ways. First of all, it enlarges the literature on the status of the Romanian health care system, by providing additional empirical evidence on the economic dimensions of COVID-19 and prospective implication on the Romanian health care system, and examines very detailed data on government policy and engagement linked to social distancing measures implemented, normative acts adopted and the areas of intervention. However, whilst examining very detailed data on government policy and budget rectifications, we also uncover an important dimension of existing limited space to propose and implement economic policy. 

The details of these methodological approaches and results of this paper are provided in the following way: Section 2 presents a retrospective analysis of Romanian public health care system and prospective implication of COVID-19. Section 3 presents materials and methods. Section 4 provides the empirical results. Section 5 discusses the results. Section 6 concludes the study.

## 2. Retrospective Analysis of Romanian Public Health Care System and Prospective Implication of COVID-19 on the Economy

In Romania, until the enforcement of the Law on Social Health Insurance no. 145/1997, the health care system was centrally coordinated by the Ministry of Health through the 41 county health directorates and the health directorate of Bucharest, consisting of a network of hospitals, polyclinics, dispensaries and other health units. Thus, until 1997, a national health system operated based on the collection of funds through taxes. In addition, there were a number of hospitals, institutes and national centres of high specialization directly subordinated to the Ministry of Health, as well as parallel medical networks, subordinated to the Ministry of Transport, Ministry of National Defence, Ministry of Labour and Social Protection and the Romanian Information Service, which provided medical services and was responsible for health care for a certain category of the population. The Law on Social Health Insurance no. 145/1997 marked the transition to a new system, namely the social health insurance system that actually started operating only in 1999, being repealed by two other normative acts that have followed one another in time, respectively the Government Emergency Ordinance no. 150/2002 on the organization and functioning of the social health insurance system, and Law no. 95/2006 on health care reform. Law no. 95/2006 is intended to represent a unitary regulatory framework for the entire health sector in Romania, being currently the legislative basis of both the social health insurance system and the entire health system.

Even if the effort to decentralize the health services system and the entire process of health resources distribution, starting from 1990 until Romania’s entry into the European Union on 1 January 2007, required new strategic plans which assured equality in health care distribution, there is still a big problem with access to health services, especially for the poor population. However, Romania’s Social Health Insurance (SHI)-based system has remained highly centralised despite recent efforts to decentralise some regulatory functions [6]. The main sources of revenue for health are social health insurance funds, supplemented by the state budget funds.

**Figure 1:**
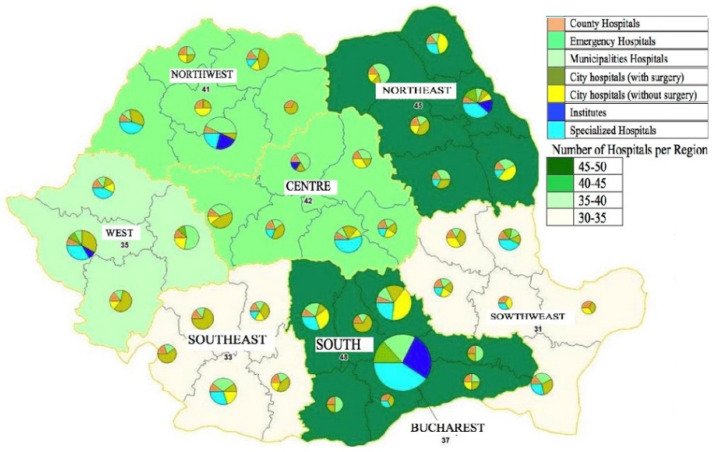
The number of Hospitals in Romania. Source: Government Decision no. 303/2011 [12]. Note: Categories of hospitals are detailed in Appendix A.

The COVID-19 pandemic put the Romanian public health system face-to-face with chaos and lack of organization, both among medical teams and patients, lacking financial support and hospital protocols. The chronic underfunding of the last decades is felt especially now, when Romania is not bypassed by the health crisis generated by the new coronavirus. Hospitals are situated, in general, in regions and large cities (see Figure 1) where there is high concentration of infrastructure and specialised medical personnel compared to the needs of people, both those infected with COVID-19 and those suffering from other diseases, especially chronic. This high concentration in urban areas limits the access of a large part of the population to quality health services, with travel time and distances exceeding optimal European level values [7]. The literature has identified the deficiencies [7,8,9,10,11] and we also emphasize the fact that the endowments are, in many cases, precarious, the medical staff is sometimes insufficient, many doctors and nurses following, over the years, the path of the foreigner in search of decent working conditions.

Given the volatility that characterizes the situation of the COVID-19 pandemic, both locally and in Europe or globally, it is vital to constantly inform and monitor the evolution of the pandemic and the measures imposed by the authorities, as well as the indications provided by them.

Because of the budgetary space, Romania has and will have major problems in the health context, being the only one in Europe that, prior to the onset of the pandemic, entered the excessive deficit procedure, with limited space for economic policies and real challenges to the sustainability of public finances (current account deficit of 4.6% of GDP in 2019 and structural budget deficit of more than 4% of GDP).

As can be seen in Table 1, in response to the COVID-19 crisis, Romania has adopted 44 normative acts, of which 14 are in the fields of economics and taxation, four are in the field of health and the others are in areas of labour and social protection, public order, public safety, education, European funds and justice. In order to cope with the additional attributions created by the COVID-19 pandemic, the Ministry of Health had at its disposal amounts from both the budget initially approved by Law no. 5/2020 of the state budget for 2020, as well as additional funding from the state budget, as a result of the rectification of the state budget (RON 1005 million), from the Budget Reserve Fund available to the Government (RON 1008.5 million), from the use of an World Bank loan (RON 85.121 million), as well as from donations and sponsorships [13]. The amount of RON 139.917 million was allocated through the National Program for Surveillance and Control of Priority Communicable Diseases [13].

Following the European Commission report (2020a), it is revealed that spending due to COVID-19 related measures, including employment support schemes and health-related spending, will put pressure on the budget, as can be seen in Table 1. In this context, budgetary space is driven by changes in overall general government balance projections. Given the past fiscal slippages and following the level of the cyclically adjusted budget balance, which in 2020 is −8.6% of potential GDP, with a predicted level of −9.9% in 2021, uncertainty remains very high in line to budgetary space for health care.

In order to manage the excess demands on the Romanian health system, it is important to maintain adequate funding for health and to correlate the lockdown measures with consumer spending, by taking into account the specifics of the income balance. The prospective implication of COVID-19 refers especially to budgetary space, which will dictate the context of urban health expenditure and will reveal not only the inconsistency in fiscal approach but also the health chronic underfunding of the last decades. 

## 3. Materials and Methods

The status of the health system in Romania can be explained with the help of a sustainability index for public health. In order to compute the sustainability index for public health, we used factor analysis, a method adopted in similar insights on this subject [16,17,18,19]. This method allowed us to estimate the overall progress of Romanian public health, over the period 1985–2019, by avoiding the problems of skewed distribution, creating independent components, and excluding the orthogonal relationship between components. All the data used in this study were created through the integration of national and international reliable sources, such as the World Bank (2020) [20], World Health Organization (WHO) database (2020) [21], European Commission database (2020b) [22] and the Romanian Ministry of Finance (2020) [23]. 

With respect to variables used in computing the sustainability index for public health, based on literature validation, we argued and established the contribution of each variable to the status of health. Health expenditures per capita (HE) represent the most suitable indicator, which provide better information in line of policy implication of changes in health care policy and, based on literature insights, a higher level of spending in this area can lead to better health results for individuals [24]. Moreover, according to Kawabata [25], the entire community financing schemes directly impact the protection of households from impoverishment. Some theoretical insights, such as those of van den Heuvel [26] and Linden [27], validate the relationship between health care expenditures and health care outcomes, such as life expectancy and mortality, which is why we included in the analysis the life expectancy at birth (LE), total (years), mortality rate (MRB), and infant (per 1000 live births). 

On the other hand, health resources in terms of physicians (PH, per 1000 people) and hospital beds (HB per 1000 people) represent important variables to demonstrate national achievements in health. In addition, they indicate not only the government policy implication in consolidating health capacity, but also the availability of inpatient services, which why we included them in the analysis. According to Scheffler [28], the policy intervention in the health area and the measures to discourage physician’s migration can affect health care services. 

Due to the fact that some papers validate the causality relationship between health expenditure, environment protection and economic growth [29,30,31,32,33], we also included in our analysis the status of real GDP per capita (as a proxy for economic growth) and some proxy for environment protection, such as CO_2_ emissions metric tons per capita, Nitrous oxide emissions (NOE), thousand metric tons of CO_2_ equivalent, GH—total greenhouse gases (including carbon dioxide (CO_2_), methane (CH_4_), nitrous oxide (N_2_O), hydrofluorocarbons (HFCs), perfluorocarbons (PFCs), sulphur hexafluoride (SF_6_) and natrium trifluoride, NF_3_) and NO—nitrous oxide (CO_2_ equivalent) at a thousand tonnes total (excluding memo items). In addition, according to Samakovlis [34], there is a direct relationship between morbidity and air pollution, thus it is appropriate to use these quality standards for the environment.

Further, the percentage of the population using improved sanitation facilities (ISFs) represent an important indicator of sustainable development goals and it is causally linked to the environmental health projects [35,36], thus it is appropriate to be used in computing the sustainability index for public health. 

Following the literature insights, which suggests that there is a direct relationship between the status of urban living and population health [37,38], and given that in Romania, due to regional disparities and to institutional competencies in area of health, social and health services are more available in cities than they are in rural areas, the last variable included in the analysis was named urban population (UP, % of total population) and represents the status of cities living and health care services. 

To estimate the sustainability index for public health (see Table 2), the percentage of the variance of each of the factors displayed in Table 3 was used and then the standardization procedure suggested by van Eck and Waltman [39] was applied, see Equation (1).

(1) $$M=\frac{\sum_{t=1}^{n}W_{t}*V_{t}}{\sum_{t=1}^{n}W_{t}}\;\;Z_{ij}=\frac{x_{ij-{\bar{x}}_{j}}}{s_{j}}$$

*M* = average value
*V_t_* = actual value for for specif
time/period *t*
*W_t_* = weighting factor for specific time/period *t*
*n* = number of periods in the weighting group
*Z_ij_* = standardized value for variable *j* in sample uniti
*x_ij_* = data for variable *j* in sample uniti
${\bar{x}}_{j\;}$= sample mean for variable *j*
*s_j_* = sample standard deviation for variable *j*

In order to study the causal relationship between health expenditure and GDP in Romania between 1985–2017, as a first step, we applied the unit root test and we found that the variables were not stationary at level. Then, the panel cointegration between the macroeconomic variables used in the model (logarithm) of health expenditure and of real GDP per capita was assessed by using the Johansen test. The process of long run co-movement between these two variables was determined based on the theoretical approach that outlines the basic features of unit root tests [40,41] for augmented Dickey Fuller (ADF) and Phillips and Perron [42] and Russell and MacKinnnon [43] for Phillips-Perron. According to the augmented Dickey Fuller (ADF) test, the methodological approach is based on the following rationale (Equation (2)):

(2) $${𝓎}_{t}=\rho {𝓎}_{t-1}+{𝓍}_{t}^{\prime }\delta +\epsilon_{t}$$

where *x*_*t*_ consists of constant, or a constant and trend, *p* and *δ* represent the parameters to be estimated, and *ϵ*_*t*_ indicates the prototypical stationary series. The null hypothesis test if H_0_: *ρ* = 1, the alternative implies that H1: *ρ* < 1. The Phillips-Perron test builds the Dickey Fuller test, whereby the null hypothesis *ρ* = 1 in ∆*yt* = (*ρ* − 1) *y*_(*t* − 1)_ + *ϵ*_*t*_, in which case ∆ represents the first difference operator. As for the implication of the Johansen test on co-integration between health expenditure and economic growth, the level data *y*_*t*_ was analysed by taking into account three deterministic trend cases:

1. It is not included the intercept $H_{2}\left(r\right):\pi_{Y_{t-1}}+B{𝓍}_{t}=\alpha \beta^{,}\pi_{Y_{t-1}}$;
2. It included intercept $H_{1}\left(r\right):\pi_{Y_{t-1}}+B{𝓍}_{t}=\alpha \beta^{,}\pi_{Y_{t-1}}+\rho_{0}$;
3. It included intercept and trend $H\left(r\right):\pi_{Y_{t-1}}+B{𝓍}_{t}=\alpha \left(\beta^{,}{𝓎}_{t-1}+\rho_{0}+\rho 1^{t}\right)+\alpha_{1}(\gamma_{0}+\gamma 1^{t)}$.

## 4. Empirical Results 

### 4.1. The Sustainability Index for Public Health

In order to estimate the overall progress of Romanian public health over the period 1985–2019, based on factor analysis methodology, we computed the sustainability index for public health (see Table 2 and Table 3). 

After we performed the factorization of the above-mentioned twelve variables, validated by literature for assessing the status of health sustainability, the results displayed in Table 2 reveal that most of the factors have an eigenvalue greater than zero and are meaningful for the analysis. Table 3 indicates the factor loadings for how the variables are weighted for each factor and reveal the proportion of the common variance of the variable not associated with factors, named the uniqueness. Table 2 and Table 3 reveal that the three main factors obtained explain the evolution of the indicators used in our analysis. Factors 1 and 2 explained 70% of the total variance and all three factors explained almost 90% of the total variance (see Table 3).

**Figure 2:**
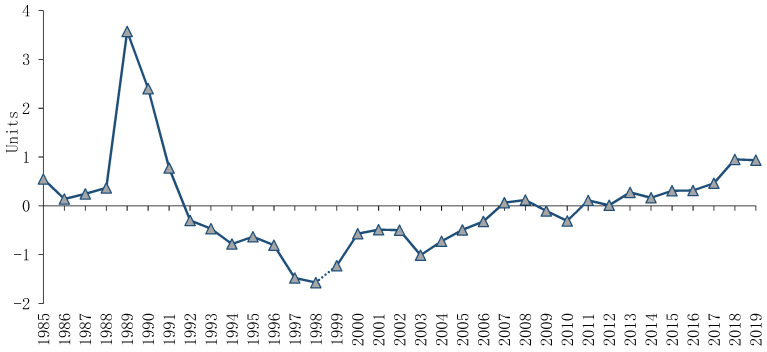
Sustainability index for public health. Source: Computed by authors processing data using Stata 15.0.

Our approach was orientated towards the empirical investigation on the Romania profile and, although there are a number of studies that review the Romanian health system [7,8,9,10,11], our analysis is differentiated by creation of a sustainability index for public health in relation to economic and environmental policy, to reveal the status of public health in line of the prospective implication of COVID-19. As can be seen in Figure 2, the study brings into focus interesting results, which confirm that the effort to decentralize the health services system and the entire process of health resources distribution still requires strategic plans and coordination mechanisms in relation to local and urban areas. Following the literature insights [44,45,46], if we talk about the implication of composite index, when the factors are a mixture of positive and negative loadings, the output could be either negative or positive, meaning that positive values are related to the consolidation process or good strategies in the analysed subject and the negative trend is specific to poor results and uninspired mechanisms in terms of policy coordination. 

According to Figure 2, the sustainability index for public health shows strong fluctuations in the period under analysis, 1985–2019. We have positive values of the sustainability index for public health before 1989, under a Soviet-type Semashko model [47] of the Romanian health system, respectively a centralized health care system, publicly funded and state-owned, with fully centralized decision-making power and with no differentiation between beneficiaries/providers in terms of service provision [48], seeking to achieve a high level of equity [47]. The major health reforms of 1989 concur with the highest level of the sustainability index for public health and bring into focus contradictory results. The results show negative values for the periods of time 1990–2006 and 2009–2010. Positive values are revealed for the periods of time 2007–2008 and 2013–2019. The results confirm the hypothesis stated by Li (2014) [49], which reveal that state policy directly affects health outcomes and depends on geographical variation and by proximity to medical services. In other words, the urbanization requires new mechanisms of coordination and strategies capable to assure the equality in line with the access to health care services [50,51].

### 4.2. The Causal Relationship between Health Expenditure and GDP in Romania 

Due to technological progress and all the reforms at the government level, in the last three decades, research on economic growth has experienced a boom and, even if in the topic of growth the interplay between health and economic growth has been a low priority, the recent literature indicates that health expenditure can be a predictor of economic growth [52,53,54,55]. 

According to the latest data published by European Commission and OECD (2018) [56], Romania ranks last among EU countries in terms of the amount spent on health as a percentage of GDP. Therefore, as the government fiscal position, and all the factors that lead to the success or failure to manage the challenges and vulnerabilities faced by the COVID-19 epidemic, depends on government solvency, it seems really appropriate to reveal the interconnection between health and the status of economic growth, this study being among the first that addresses this approach on Romania’s profile. 

Given that our approach is orientated towards the causal relationship between health expenditure and GDP in Romania as a first step, we apply the unit root test. Based on data displayed in Table 4, the results for both the augmented Dickey Fuller (ADF) test and the Phillips and Perron test indicate that the null hypothesis cannot be rejected, and we assume that our variables are nonstationary. Even if on the profile of health expenditure per capita (HE) the t statistics is −3.96, and it is it is weekly significant at 10% because we are basing it off a significant level of 5%, we cannot reject the null hypothesis that the log of HE has a unit root.

The methodological approach avoids the implication of spurious regression, which means that we follow the theoretical literature that suggests that for non-stationary variables we should always think in terms of cointegration [57,58,59]. Therefore, in Table 5, we employed the Johansen test on co-integration between health expenditure and economic growth. Following the literature insights that suggest that the cointegration test should be performed on the level form of the variables, we considered it appropriate to use the log transformation of the variables and we assumed two decision criteria: Rejection at the 5% level and reject the null hypothesis if the value of the trace and max statistics is greater than the 5% critical value, otherwise, fail to reject the null hypothesis. The none implies that there is no cointegrated equation in the model and, looking to our results, Table 5 indicates that the trace is greater than the 5% critical value and the probability value is lower than 1% (0.0088), which means that we reject the null hypothesis that there is no cointegrated equation in this model. The second hypothesis reveals that there is at most one cointegrated equation, whereby the trace value (2.785185) is lower than 5% of the critical value (3.841466). Following the Max-Eigen statistics, our decisions are not different to what we meet on trace statistics. The Max-Eigen statistics is 18.83810, which is greater than the 5% of the critical value (14.26460) and reveals that we reject the null hypothesis at the 5% level. For the second null hypothesis of at most 1, we can reject the null and we agree that there is at most one cointegrated equation.

The existence of cointegration between health expenditure and economic growth, exhibit a causal relationship and reveal the importance of consolidate the efficiency of health spending system. The results confirm the health-led growth hypothesis, which is validated in the work of Atilgan [61], and reveal that the increase in health expenditure and the efforts to unifying all health insurance schemes could lead not only to improvement in health services but also to economic performance. 

## 5. Discussions

### 5.1. The Sustainability Index for Public Health

The results of the sustainability index for public health reveal that even at the beginning of the 1990s, the Romanian government has tried to change the way they are provided the services within the process of modernization of the Romanian society and preparing the accession to the EU.

Based on the sustainability index for public health dynamic over the period 1985–1989, according to literature [62], Semashko systems provided free access to health care and focused on curing acute rather than chronic diseases. The system was based on hospital and specialized health care and health publishes on a large scale, on top-down interventions against the most singular health problems, being controlled by the state through the centralized planning system [63]. Therefore, due to the fact that, in that period at that level, the implication of urbanization was at a low level and the communist regime established that the doctors should be reached even in the most remote villages, through the distribution at the end of the faculty, people had free access to medicine and the status of health system was not centrally oriented; the population’s access to health services being equal [63]. In all the villages and communes there were dispensaries where doctors were obliged to attend by the distribution received at the end of the study, and each Romanian was assigned to a medical office or dispensary. 

In 1989 and later, during the 1998 reforms, decentralization and pluralism of service provision, compulsory social security and contractual relations between service providers and purchasers were introduced in Romania [64]. The combination of suppliers consisted of a few small private companies that provided primary health care, numerous lower, middle and upper level hospitals and a few specialized outpatient units and secondary outpatient care units (diagnosis and treatment). There was a coordination in terms of budgetary space and a limited referral system between these service levels or between units, which seems to be appropriate until 1992. However, after this period, the results reveal some deficiencies related to the health system reform implementation and the entire status of health care services; aspects identified by the literature [7,8,9,10,11]. In this context, the negative values for the period 1990–2006 are explained by the fact that, in the years after the Revolution, the system operated out of inertia and even if the new reform establishes the institutional mechanism in the area of health, the Health Insurance House (aimed to collect contributions from employees) does not improve the health care system. In this context, the system found itself underfunded and incapable of satisfying citizens’ interests. 

The positive results from the year 2007 and during the period 2013–2019 reveal, on the one hand, Romania’s entry into the European Union, on 1 January 2007, which required new strategic plans in assured equality in health care distribution. Numerous hospitals have been upgraded, the supply has increased emergency services and the services provided by private entities have been developed. Separation of health care providers of service providers and the introduction of relationship contractual agreements between them was another major component of the modernization process. The period 2013–2019 indicates the constant reform in health services, including a new type of financial resources management based on the initiative to create the new administration within the hospital sector. 

Romania’s profile of the health status of Romanians has improved, but life expectancy at birth remains among the lowest in the EU. An important issue is access to health care, which is particularly low in rural areas and is exacerbated by differences in demographic coverage, which makes unmet health care needs substantially above the EU average and requires new mechanism of strategies in terms of budgetary space.

### 5.2. The Causal Relationship between Health Expenditure and GDP in Romania 

The results of the study reflect the implication of the COVID-19 pandemic on the Romania budgetary mechanism established to cover public health expenditure, confirming the importance of understanding the sources of vulnerabilities that can lead to a financial crisis but also validate the health-led growth hypothesis, and indicate that the prospective implication of COVID-19 depends on budgetary space which will dictate the efficiency of health expenditure. The study confirms that Romania has some problems in understanding the status of budgetary space. The fact should be accepted that an increase in health expenditure and the efforts to unifying all health insurance schemes could lead not only to improvement in health services but also to economic performance. 

After 1990, the social health insurance system is characterized by centralization in health resources distribution and all the policy guidance and regulatory oversight comes from the national level (Ministry of Health). Despite recent efforts to decentralize some regulatory functions, the local authorities remain responsible for the delivery of services but depend on national guidelines. In summary, the efficiency of the Romanian health system depends on economic and policy stabilization, but also on the effort to decentralize the health services system and the entire process of health resources distribution. Additionally, it seems that the idea of centralization is found not only at this level. A study based on the Romania profile revealed that after 1990 a perfect fiscal centralization was approached and starting with 2006, due to the new legislative requirements, a relative fiscal centralization characterized Romanian local authorities [65].

Competences in the health area and all the money are delegated especially to hospitals in urban areas (see Figure 1), which has low implications on rural areas. The health system is essential for ensuring a high level of cohesion and social protection in Romania. Variations at the institutional level, differences in organization and incorrect management of financial resources prevent access to quality care and deprive citizens of what equity or solidarity means. That is why our analysis presents important prospective insights related to government policy and engagement linked to social distancing measures implemented, budget rectifications and normative acts adopted. In addition, the areas of intervention in the time of the COVID-19 pandemic emphases not only the status of Romanian health sustainability or the health-led growth hypothesis, but also the direction to follow and the institutional vulnerabilities related to the health care system.

Applying the unit root test assesses the causal relationship between health expenditure and GDP in Romania between 1985–2017, and we find that the variables are not stationary at level. Based on the Johansen test on co-integration, the existence of cointegration between health expenditure and economic growth is revealed, thus confirming the health-led growth hypothesis. The overall progress of the Romanian public health system over the period 1985–2019 is revealed by the results of factor analysis methodology, which help us to compute the sustainability index for public health. The factorization of selected and theoretically-validated variables reveal that most of the factors have an eigenvalue greater than zero and are meaningful for the analysis. The three main factors obtained explained the evolution of the indicators used in our analysis. Factors 1 and 2 explained 70% of the total variance and all three factors explained almost 90% of the total variance. 

The empirical investigation on the Romanian health system confirms that the effort to decentralize the health services system and the entire process of health resources distribution still required strategic plans and coordination mechanisms in relation to local and urban areas. 

In summary, the study reveals the imperfect status of Romanian budgetary space. It seems that the government fiscal position and vulnerabilities could not assure the capabilities to manage the excess demands on the Romanian health system, to maintain adequate funding for health and to correlate the lockdown measures with consumer spending, by considering the specifics of income balance. The fact should be accepted that an increase in health expenditure and in the efforts to unify all health insurance schemes could lead not only to improvement in health services but also to economic performance, this being confirmed by the health-led growth hypothesis.

Even if the sustainability index for public health methodology and the Gregory-Hansen cointegration method are powerful tools for, on the one hand, emphasizing the status of the health system in Romania and, on the other hand, to provide new evidence on the causal relationship between health expenditure and GDP for Romania, our analysis has some limitations regarding the data available for a longer period of time and closer to the present. 

## 6. Conclusions

This study has successfully answered the research paper aims: (1) To highlight the status of the health system in Romania, explained with the help of an own-computed sustainability index for public health; and (2) to provide new evidence on the causal relationship between health expenditure and economic growth for Romania using the Gregory-Hansen cointegration method. From the path analysis results, it was found that the sustainability index for public health confirmed that the effort to decentralize the health services system and the entire process of health resources distribution still required strategic plans and coordination mechanisms in relation to local and urban areas. However, the system remains highly centralized and the chronic underfunding of the last decades is felt especially now, when Romania is not bypassed by the health crisis generated by the new COVID-19 pandemic. Second, following the existence of the causal relationship between health expenditure and GDP for Romania, we confirm the health-led growth hypothesis, and we reveal that an increase in health expenditure and the efforts to unify all health insurance schemes could lead not only to improvement in health services but also to economic performance. Finally, the findings indicate that the process of upgrading the health system will put pressure on government budgets, and the prospective implication of COVID-19 refers especially to budgetary space. The results are relevant for guiding policy makers to be interested in effectiveness in rural and urban health management, as well as to improve the overall general government balance projections. Therefore, taking into consideration the above-mentioned background, it is required to mention the government policy and engagement linked to social distancing measures implemented, budget pressures, normative acts adopted and the areas of intervention, with the objective to signalize the prospective implication of COVID-19 on the Romanian health care system.

As a future research direction, we intend to extend the analysis at the European Union level, by developing the sustainability index for public health for all the European Union countries, including the ex-communist states (Central and Eastern European countries) for a comparative approach with West European states. From the perspective of future research, the causal relationship between health expenditure and GDP in the European Union countries and the comparative approach between ex-communist countries (Central and Eastern European countries) and West European states should be another next step of our work.

## Figures and Tables

**Table 1:** The impact of the COVID-19 crisis on the Romanian economy.

Indicators		Years	
	2020	2021 *	2022 *
GDP %	−5.2	3.3	3.8
Unemployment rate %	5.9	6.2	5.1
General government balance % GDP	−10.3	−11.3	−12.5
Cyclically adjusted budget balance as a % of potential GDP	−8.6	−9.9	−11.5
General government gross debt % GDP	46.7	54.6	63.6
**Normative acts adopted in the context of the COVID pandemic (2020)**			
Military Ordinance	9		
Emergency Government Ordinance	10		
Government Decision	25		

Source: European Commission (2020a) [14] and Romanian Government (2020) [15]. Note: * = Financial forecasting.

**Table 2:** The results of factor analysis of the main components for estimating the sustainability index for public health.

Factor Analysis/Correlation, Method: Principal Factors, Rotation: (Unrotated)				
Factor	Eigenvalue	Difference	Proportion	Cumulative
Factor1	7.70694	5.12482	0.6640	0.6640
Factor2	2.58212	1.90516	0.2225	0.8864
Factor3	0.67696	0.35902	0.0583	0.9447
Factor4	0.31794	0.11200	0.0274	0.9721
Factor5	0.20594	0.08267	0.0177	0.9899
Factor6	0.12326	0.10286	0.0106	1.0005
Factor7	0.02041	0.01044	0.0018	1.0022
Factor8	0.00997	0.01200	0.0009	1.0031
Factor9	−0.00203	0.00474	−0.0002	1.0029
Factor10	−0.00677	0.00431	−0.0006	1.0023
Factor11	−0.01108	0.00506	−0.0010	1.0014
Factor12	−0.01614		−0.0014	1.0000

**Table 3:** Factor loading and explained variance.

Unrotated Loadings				
	F1	F2	Uniqueness	
HE	0.7813	0.1740	0.3593	
MRB	−0.9895	0.1154	0.0076	
PH	0.8383	−0.2166	0.2504	
HB	−0.9295	−0.0181	0.1357	
CO_2_	−0.8586	−0.2395	0.2054	
NOE	−0.9181	−0.1555	0.1330	
GH	−0.0434	0.9690	0.0592	
NO	0.1578	0.9644	0.0450	
ISF	0.8800	−0.2825	0.1458	
UP	0.6725	0.5815	0.2097	
LE	0.9545	−0.2361	0.0333	
GDP	0.8979	−0.2591	0.1267	
Factor	Variance	Difference	Proportion	Cumulative
F1	4.63553	1.11718	0.3994	0.3994
F2	3.51836	1.11009	0.3031	0.7025
F3	1.40826	…	0.1075	0.9099

Source: Computed by authors processing data using Stata 15.0. Note: HE—health expenditure per capita, MRB—mortality rate, PH—health resources in terms of physicians (PH, per 1000 people), HB—health resources in terms of hospital beds (HB per 1000 people), CO_2_—carbon dioxide emissions metric tons per capita, NOE—nitrous oxide emissions, thousand metric tons of CO_2_ equivalent, GH—total greenhouse gases (including carbon dioxide (CO_2_), NO—nitrous oxide (CO_2_ equivalent) thousand tonnes total (excluding memo items), ISF—improved sanitation facilities, UP—urban population (UP % of total population), LE—life expectancy at birth, GDP—real GDP per capita.

**Table 4:** Unit root test results.

Variables	Deterministics	ADF		PP	
		t-stat	prob	t-stat	prob
Log real GDP per capita	None	2.95	(0.839)	2.03	(0.988)
	Intercept	0.54	(0.592)	0.73	(0.467)
	Intercept and trend	−1.13	(0.264)	−2.33	(0.407)
Log health expenditure per capita (HE)	None	1.93	(0.985)	1.69	(0.976)
	Intercept	0.56	(0.574)	−0.66	(0.508)
	Intercept and trend	−3.96	(0.020)	−3.93	(0.021)

Null Hypothesis: LREAL_GDP_PER_CAPITA has a unit root.

**Table 5:** The Johansen test on co-integration between health expenditure and economic growth.

Unrestricted Cointegration Rank Test (Trace)				
Hypothesized		Trace	0.05	
No. of CE(s)	Eigenvalue	Statistic	Critical Value	Prob. **
None *	0.444946	21.62329	15.49471	0.0053
At most 1	0.083357	2.785185	3.841466	0.0951
**Unrestricted Cointegration Rank Test (Maximum Eigenvalue)**				
Hypothesized		Max-Eigen	0.05	
No. of CE(s)	Eigenvalue	Statistic	Critical Value	Prob. **
None *	0.444946	18.83810	14.26460	0.0088
At most 1	0.083357	2.785185	3.841466	0.0951

Trace test indicates one cointegrating equation(s) at the 0.05 level, * denotes rejection of the hypothesis at the 0.05 level, ** MacKinnon, Haug and Michelis (1999) [60] *p*-values. Max-eigenvalue test indicates one cointegrating equation(s) at the 0.05 level, *denotes rejection of the hypothesis at the 0.05 level, ** MacKinnon, Haug and Michelis (1999) [60] *p*-values.

## Data Availability

Data of the retrieved studies are shown in Appendix A.

## Appendix A

Categories of hospitals in Romania based on Law 95/2006 and Ministry of Health Classification:

- County Hospitals—the hospitals located in the capital county, which have the human and material resources and the competences to ensure definitive medical care (including emergency medical care) for most cases that come from the county and cannot be treated permanently locally in hospitals municipal or urban centres or in the permanence centres, in accordance with the protocols in force;
- Emergency Hospitals—county hospitals, municipal hospitals, city hospitals (with surgery), and specialized hospitals with human and material resources and the competences to ensure definitive emergency medical care;
- Municipalities Hospitals—the hospitals located in municipal cities, respectively to centre of permanence, with the human and material resources and the competences to ensure definitive medical care (including emergency medical care) necessary to solve a part of the local emergencies; the emergencies that cannot be solved definitively are stabilized and transferred to the county hospital, as the case may be, in accordance with the protocols in force;
- City Hospitals (with surgery)—the hospitals located in cities with infrastructure to treat locally (including emergency medical care); the emergencies that cannot be solved definitively are stabilized and transferred to the municipal or county hospital, in accordance with the protocols in force;
- City Hospitals (without surgery)—the hospitals located in cities with infrastructure to treat locally; the emergencies are stabilized and transferred to the municipal or county hospital, in accordance with the protocols in force;
- Institutes or public health centres are regional or national public institutions, with legal personality, subordinated to the Ministry of Health, and which technically and methodologically coordinate the specialized activity in the field of substantiation, elaboration and implementation of strategies regarding disease prevention, control of diseases, and of public health policies in specific fields, at national and/or regional level; if they have a university clinical department they are clinical hospitals;
- Specialized Hospitals—hospitals that treat patients mainly with a certain dysfunction or from a certain social category. Thus, this category includes burn hospitals (treats people with burns), hospitals for people with mental health problems and children’s hospitals.
